# Arthroscopic Reinsertion of an Anterior Cruciate Ligament Tibial Spine Fracture Avulsion With Double‐Strand Lacing of Anterior and Posterior Bundles

**DOI:** 10.1002/atn2.70131

**Published:** 2026-06-12

**Authors:** Matthieu Peras, Styliani Stergiadou, Inés Restrepo Garcia, Valentin Sudaros, Rayan Fairag, Jean‐François Gonzalez, Grégoire Micicoi

**Affiliations:** ^1^ Hôpital d’Instruction des Armées Sainte‐Anne Toulon France; ^2^ IULS‐University Institute for Locomotion and Sports Pasteur 2 Hospital Hôpital Pasteur II Nice France; ^3^ Department of Orthopaedic Surgery and Musculoskeletal Trauma University Hospital of Larissa School of Health Sciences University of Thessaly Larissa Greece

## Abstract

Avulsion fractures of the tibial spines have the same mechanism as anterior cruciate ligament ruptures but occur more often in subjects with an immature skeleton or in osteoporotic subjects. Displacement of more than 2 mm generally requires fixation. Several open or arthroscopic techniques have been described: from screwing of the spines to suturing of the anterior cruciate ligament. We describe in this technical note, with a video, a 4‐strand suture in the 2 bundles of the anterior cruciate ligament under arthroscopy, allowing a more anatomical and stable fixation of the fracture, without placement of intraosseous material, which can be a good option in young patients.

## Graphical abstract

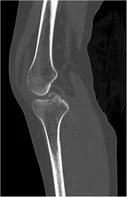

**VIDEO 1:** This video presents the arthroscopic technique of tibial spine reinsertion by double strand of the 2 bundles of the anterior cruciate ligament. The patient is positioned supine with the knee bent at 90°. The camera is inserted through the anterolateral approach. We successively observe the debridement of the fracture, the passage of the strands in the 2 bundles of the anterior cruciate ligament, the creation of the 4 trans osseous tunnels using the guide, the passage of the strands in the tunnels with the relay wire, and the tightening of the endo‐buttons with final arthroscopic control of the tension of the anterior cruciate ligament. The video ends with the radiological result 1 year postoperatively. Video content can be viewed at https://doi.org/10.1002/atn2.70131.

Although the mechanism (torsion of the knee in valgus flexion and external rotation) is identical to the rupture of the anterior cruciate ligament (ACL), the avulsion of the tibial spines occurs in patients with an immature skeleton or in osteoporotic subjects.^1^, ^2^ Meyers and Mac Keever published in 1959 a classification of tibial spine fractures.^3^ Generally, stages I and II are accessible to nonoperative treatment, and stages III and IV (displacement of 2 mm and more) are surgical. Several techniques have been described, whether arthroscopic or open surgery, ranging from single‐bundle lacing with endo‐button fixation^4^ or screwing ACL insertion in tibial spines.^5^, ^6^ Here, we describe an original technique for reinsertion of the tibial spines by lacing using 4 strands on the 2 bundles of the ACL, allowing a stable and an anatomical fixation of the fracture; it also allows a more progressive reduction, which can be useful in the case of communitive fractures.

## SURGICAL TECHNIQUE

### Indication

**FIGURE 1:**
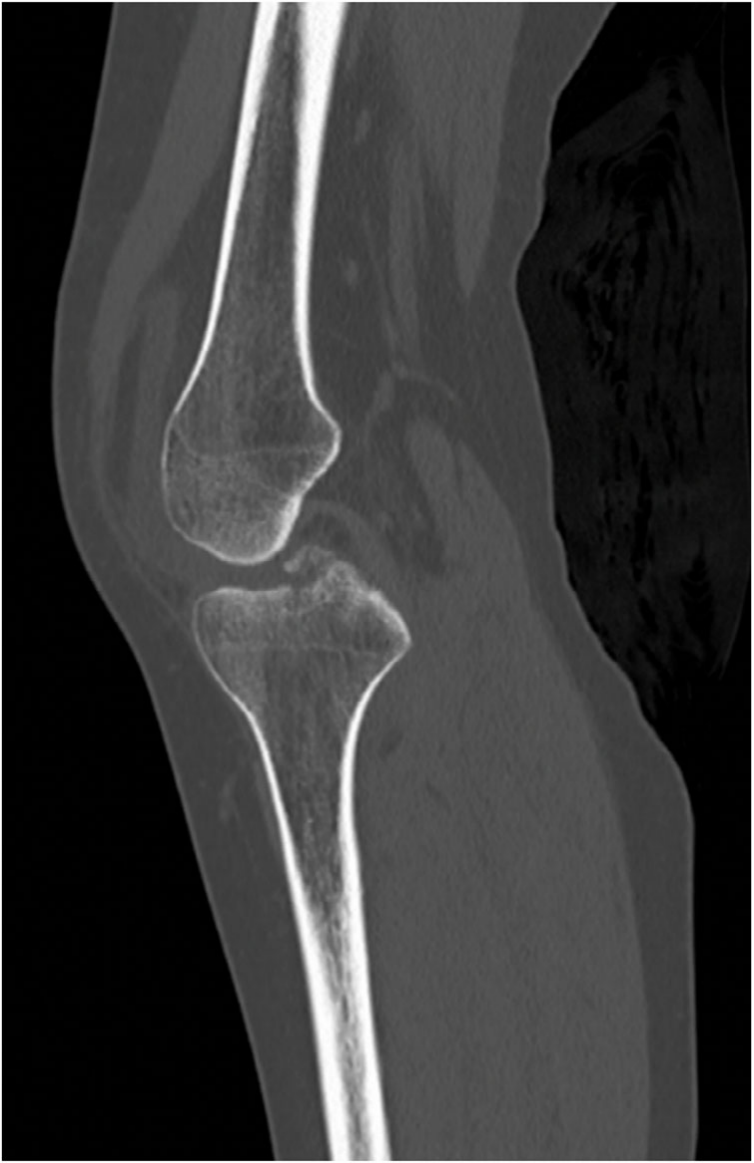
Avulsion fracture of the tibial spines with a displacement of more than 2 mm seen on sagittal section.

Surgery was indicated for avulsion fractures of the tibial spines with a displacement of more than 2 mm on computed tomography (Figure 1). A magnetic resonance imaging was systematically performed to rule out multiligament involvement of the knee.

### Surgical Preparation

Patient is placed in supine position, with the knee bent at 90°. A tourniquet is positioned at the base of the thigh and inflated to 250 mmHg during surgery. The image intensifier is used to check the correct reduction of the fracture at the end of the surgery (Video 1).

### Landmarks and Approach

**FIGURE 2:**
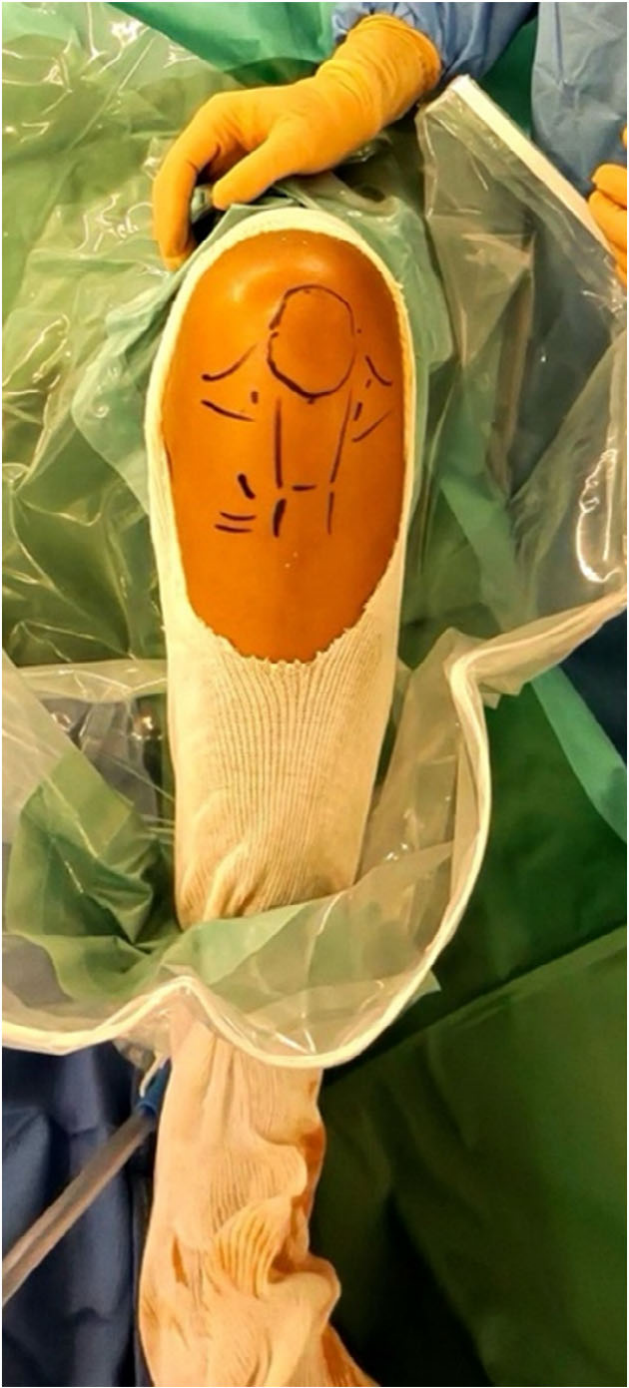
Skin landmark on a left knee, patient in supine position.

Anterolateral and anteromedial optical approaches of knee arthroscopy are used. A third high anteromedial, performed with a needle under arthroscopic control, approach is used to protect wires during drilling the tunnels (Figure 2).

### Exposure and Reduction of the Fracture

**FIGURE 3:**
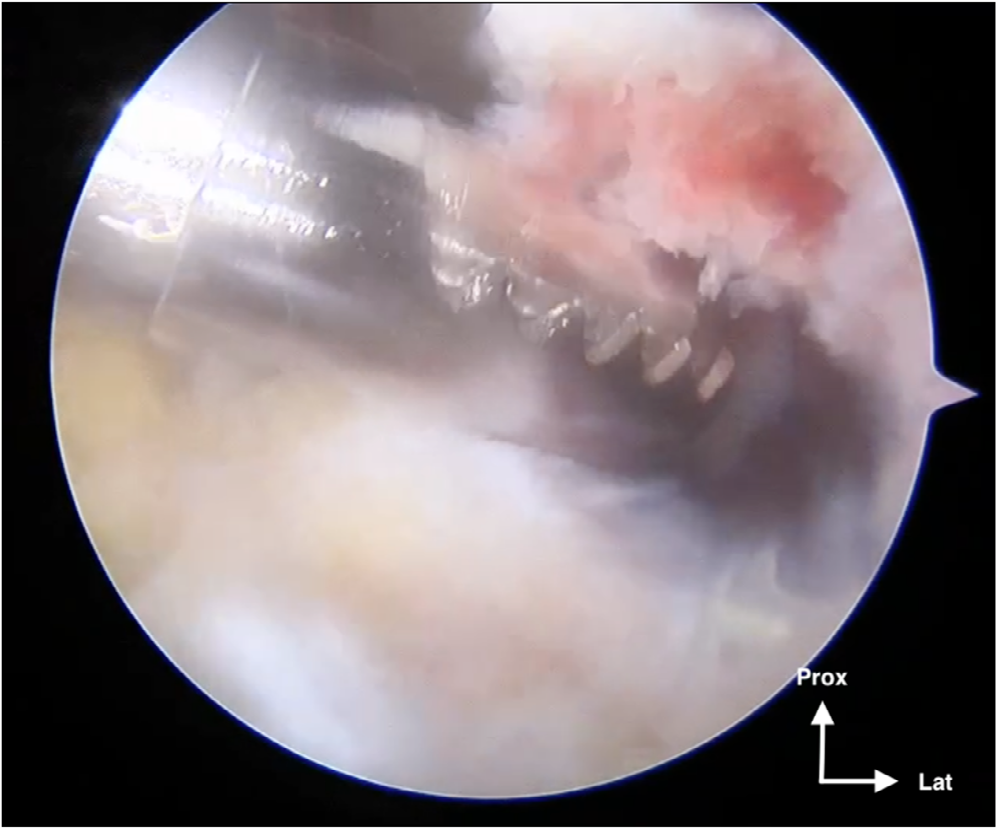
Shaver debridement of the fracture site. (Lat, lateral; Prox, proximal.)

After evacuation of the hematoma and cleaning of the fracture with the shaver, the reducibility of the bone fragment is tested using the probe. At this point, the absence of incarceration, particularly of the anterior intermeniscal ligament, is ensured with the arthroscopic control (Figure 3).

### Passing Wires and Tunnels Drilling

**FIGURE 4:**
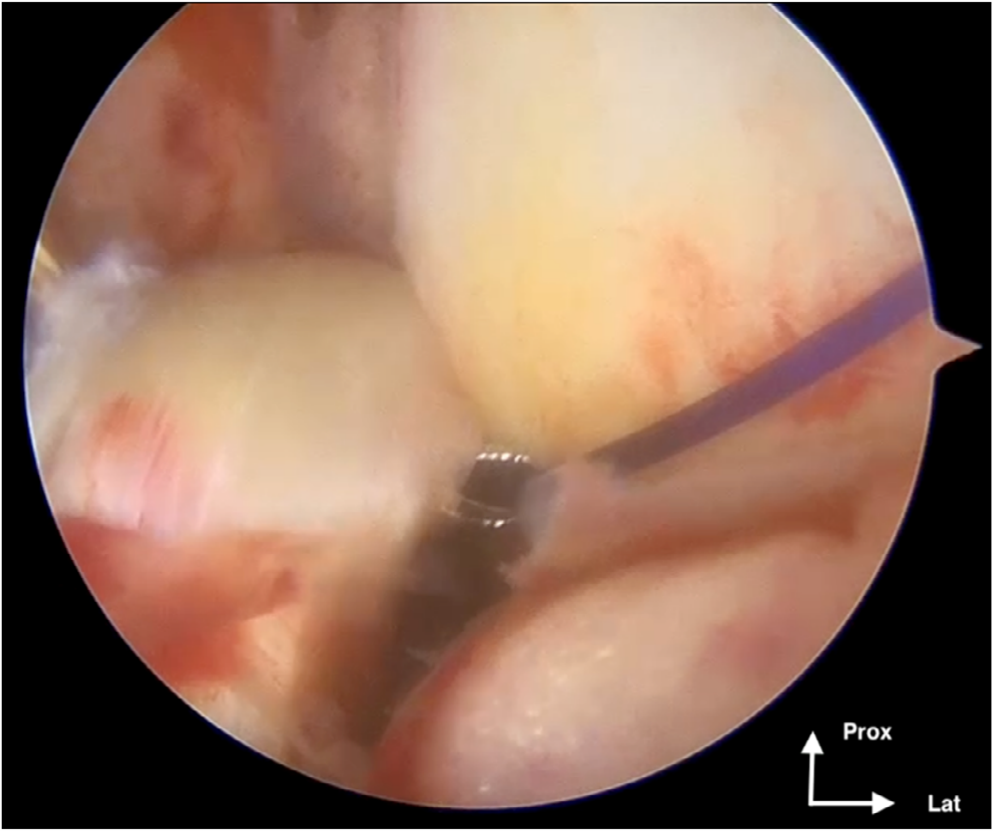
Passing spectrum in the posterior ACL bundle, with PDS suture relay. (ACL, anterior cruciate ligament; Lat, lateral; Prox, proximal.)

**FIGURE 5:**
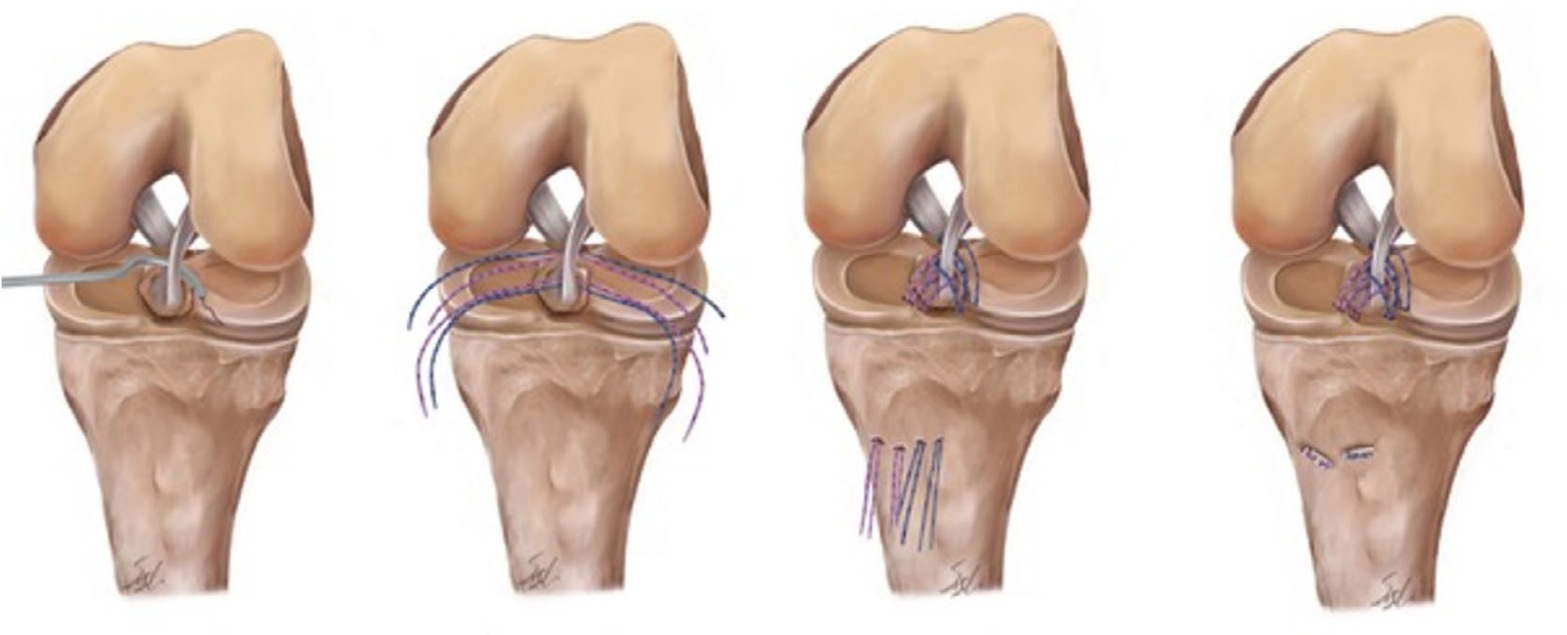
Frontal anatomical drawing of passing wires.

**FIGURE 6:**
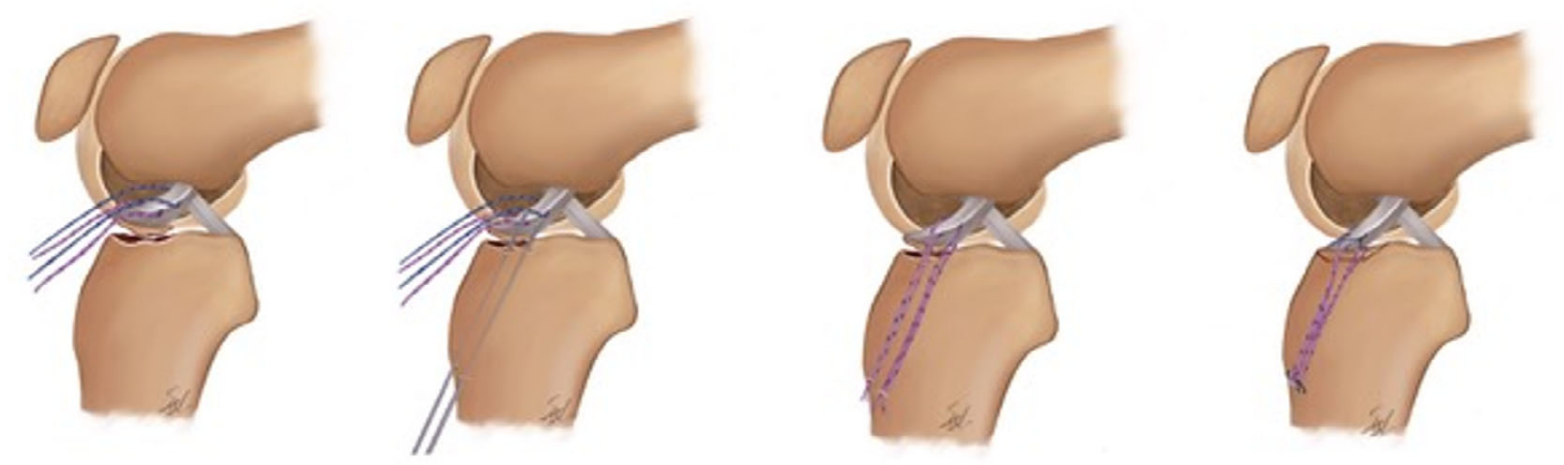
Sagittal anatomical drawing of passing wires.

First relay suture (PDS 0) is passed using the Spectrum (ConMed, Largo, FL) into the posterior bundle at the tibial insertion of the ACL (Figure 4). This first relay suture is retrieved in the high anteromedial pathway, and 2 braided wires diameter 2.0 mm of 2 different colors are passed through the foot of the posterior bundle of the ACL via the relay suture (wires P1 and P2) (Figures 5 and 6).

The second relay wire is passed using the same technique, this time in the anterior bundle of the ACL; the same operation with the relay wire is carried out in order this time to have 2 anterior strands (A1 and A2).

**FIGURE 7:**
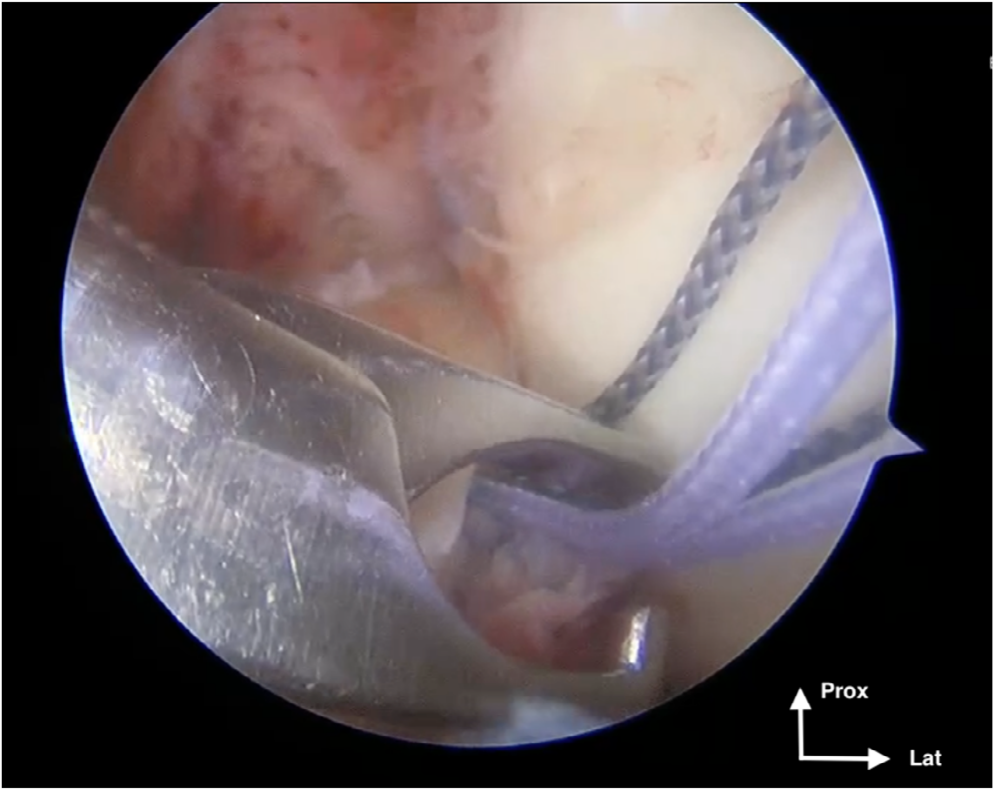
Detangling wires before passing them in the AM‐H track. (AM‐H, high anteromedial; Lat, lateral; Prox, proximal.)

We ensure that there is no snag on each strand using the detangling pliers each time the wires are passed; then, the wires P1, P2, A1, and A2 are positioned in the high anteromedial “garage” track (Figure 7).

**FIGURE 8:**
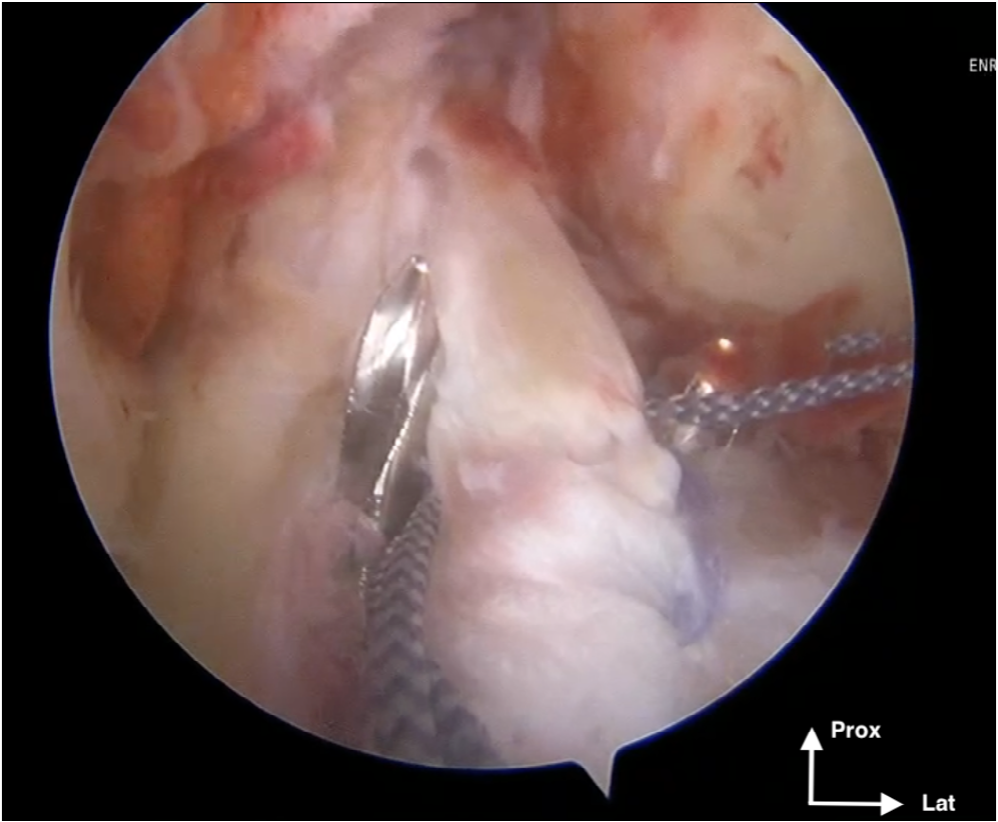
Placement of 3.0 mm pins lateral and medial to ACL in the fracture site. (ACL, anterior cruciate ligament; Lat, lateral; Prox, proximal.)

**FIGURE 9:**
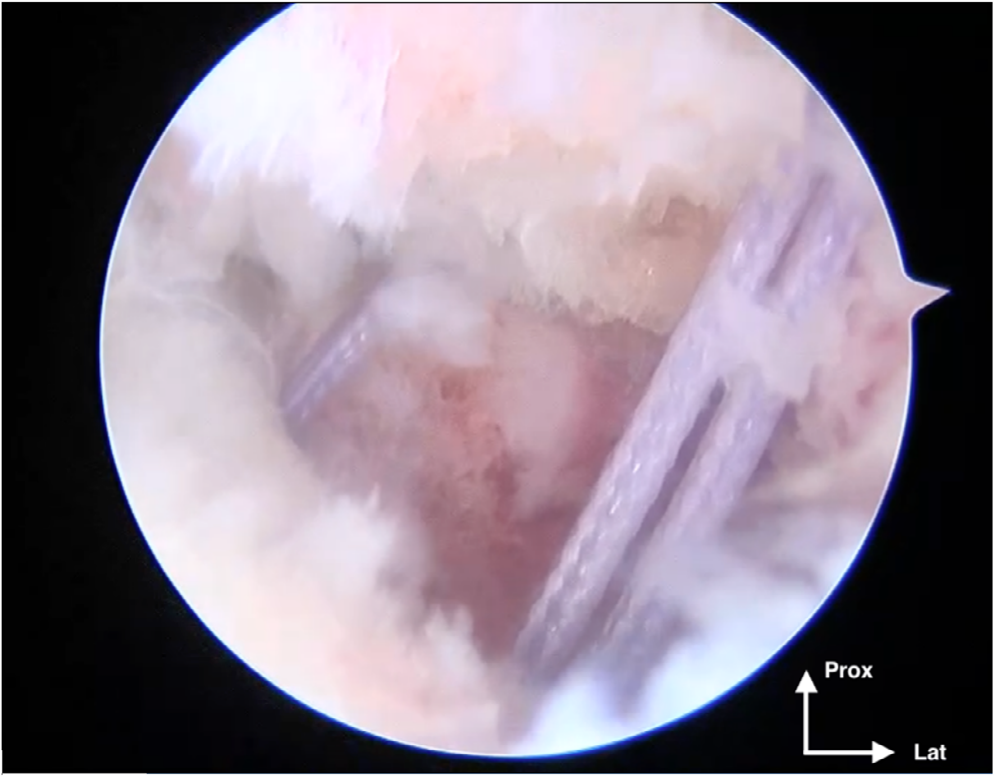
Reducing fracture pulling on strands. (Lat, lateral; Prox, proximal.)

The first 2 central tunnels are made using a 3.0 mm pin and an ACL tibial aiming device (Figure 8). Pins are placed in the tibial fracture site to create a centrolateral tunnel and a mediolateral tunnel. The first 2 wires P1 and A1 are then passed, respectively, into the ML and anterolateral tunnel using the Acufex passer (Smith & Nephew, Hertford, UK) and a relay PDS. The fracture is then reduced by pulling on these first 4 strands (Figure 9).

**FIGURE 10:**
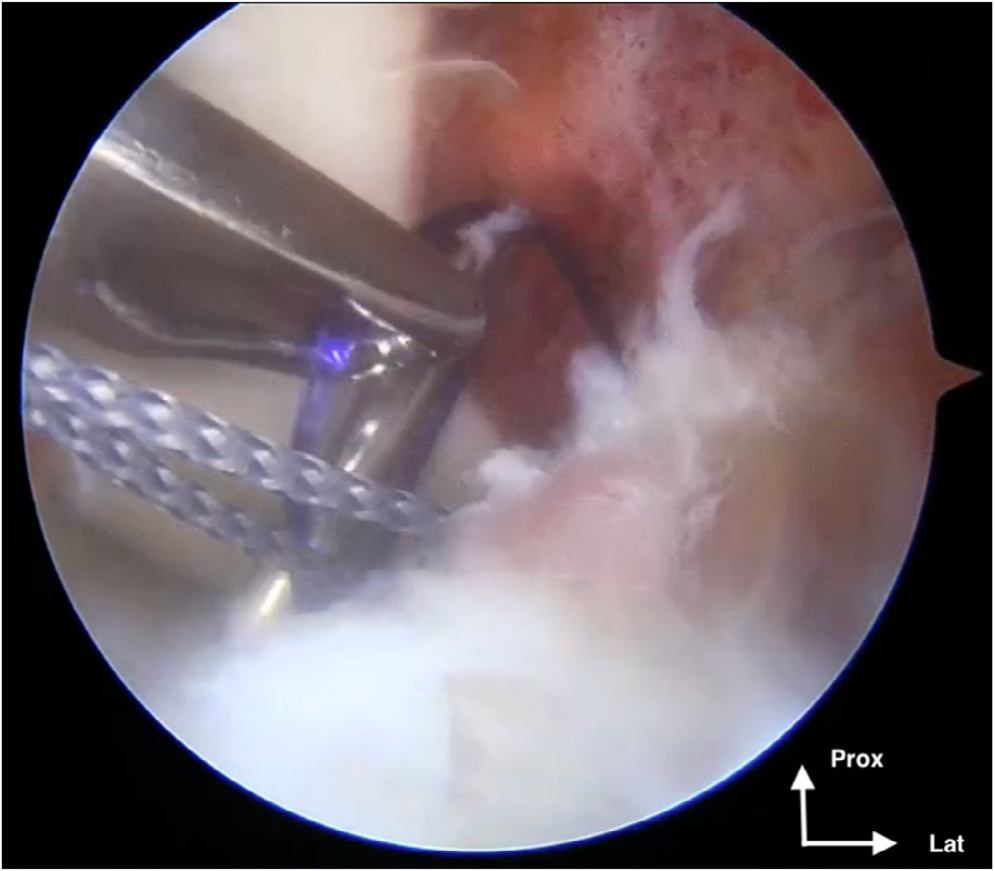
Relay PDS passing using the Acufex passer. (Lat, lateral; Prox, proximal.)

Then, the 2 peripheral tunnels are made 3 mm lateral and medial to the fracture (LF and MF), and the A2 and P2 wires are then passed into the LF and MF tunnel using the same technique (Figure 10).

Care must be taken to ensure that the entry points of the pins at the tibial cortex are not too far apart to be able to pass the strands through the 2 endo‐buttons and far enough to avoid a cortical fracture between tunnels.

**FIGURE 11:**
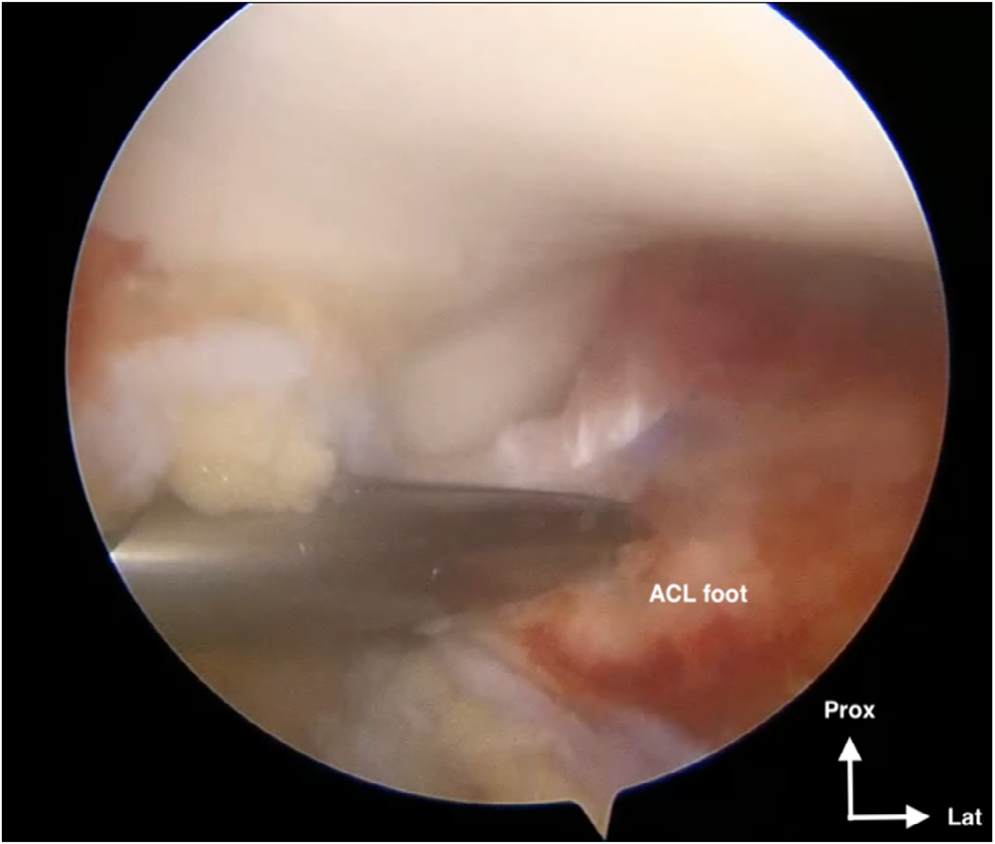
Reduction using the probe while knots are tightening. (ACL, anterior cruciate ligament; Lat, lateral; Prox, proximal.)

**FIGURE 12:**
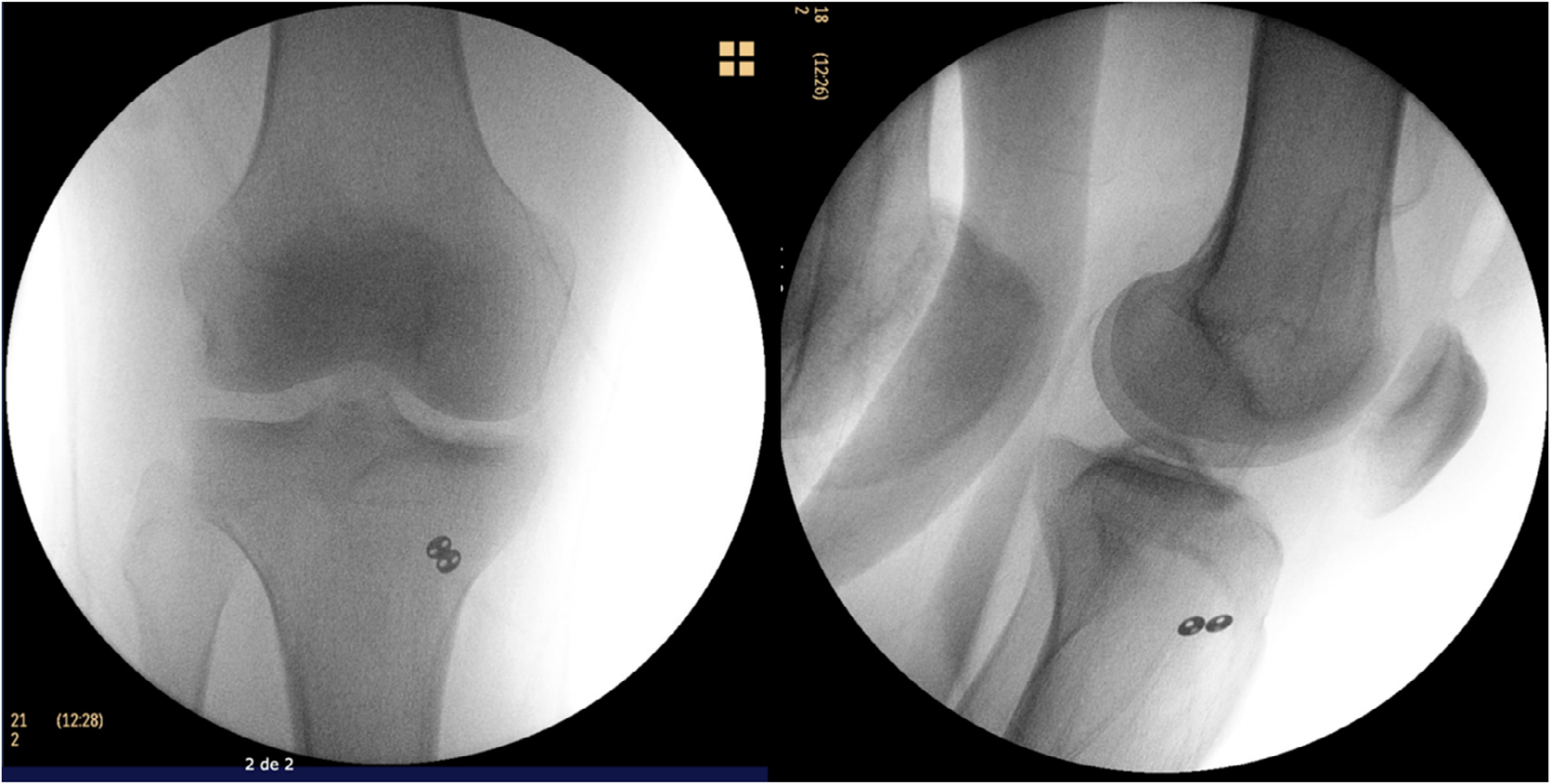
Final scopic controls. We can observe the good reduction of the tibial spine as well as the good placement of the endo‐buttons.

Finally, each strand is fixed in its corresponding button (lateral or medial) by tightening the knot at 30° flexion, with the probe on the tibial spine to control intra‐articular reduction (Figure 11). A final scopic control (Figure 12) is carried out to ensure the correct placement of the implants and fracture reduction.

### Postoperative Care

Partial weight‐bearing on the operated limb is permitted, and the patient is immobilized in an articulate knee brace in extension. Physiotherapy starts immediately with a progressive range of flexion: 0° to 45° for 2 weeks, then 0° to 60° for 2 weeks, then 0° to 90° for 2 weeks, and after 6 weeks, full range of flexion is permitted. Patients are reviewed at 45 days, 3 months, 6 months, and 1 year postoperatively with follow‐up radiograph.

## DISCUSSION

We describe a minimally invasive technique performed under arthroscopy with an anatomical reinsertion of the 2 ACL bundles, each fixed by 4 strands.

This technique may seem more difficult to perform than simple lacing or other techniques, mainly due to the need to make 4 tunnels of the wire diameter; however, it allows the treatment of simple and comminuted fractures with an anatomical repositioning of the 2 ACL bundles. In addition, performing the technique arthroscopically allows for a meniscal procedure in the event of an associated lesion.^7^ Advantages and disadvantages of other techniques available in the treatment of tibial spine fractures are summarized in Table 1.

**TABLE 1:** Advantages and Disadvantages of Different Surgical Techniques

Technique	Advantages	Disadvantages
Reinsertion with transosseous tunnels (presented technique)	Available on comminuted fractures Poor discomfort from buttons	Difficult technique Tunnel passing through the physis in immature skeleton
Screwing	Simple procedure Compression of the fracture site	Difficult on comminuted fracture Screw removal is necessary if there is a conflict with the trochlea
Suture on anchor	Simple and possible procedure on comminuted fractures	Biomechanically weak fixation

Most published studies on other techniques primarily involve the pediatric population, with comparatively fewer studies focusing on adults. In adults, the literature review by Salvato et al. in 2023 shows that arthroscopic techniques allow earlier recovery than open techniques, regardless of the means of fixation used.^1^ Our clinical and radiological results are in agreement with the meta‐analysis by Osti et al., published in 2016.^8^

We did not have any pseudarthrosis of the tibial spines in the postoperative follow‐up although this has been described in rare cases.^9^, ^10^ In this case, our technique does not prevent an ACL reconstruction.

The main limitation of our study is the small number of patients due to an indication that remains relatively rare in practice. We also do not have a biomechanical study supporting our theory of increased stability of the 4‐strand reinsertion.

Our technique is reproducible, with little risk of complications and gives correct functional results postoperatively. Certain critical aspects and potential pitfalls must still be considered during surgery and are summarized in Table 2.

**TABLE 2:** Pearls and Pitfalls of Double‐Strand ACL Lacing

Pearls	Pitfalls
Take the time to wash hemarthrosis and clean fracture site	Cutting wires while drilling the tunnels, ensure proper strands placement in the AM‐H approach
Tighten strands with probe pressure on tibial spine for a good ACL tension	Do not untangle threads and do not check for capsular incarceration before retrieving them in tunnels
Do not hesitate to over‐reduce tibial spine fragment to achieve good ACL tension	Drilling tunnels too close and cause a cortical collapse

ACL, anterior cruciate ligament; AM‐H, high anteromedial.

## DISCLOSURES

The author (J‐F.G.) declares the following financial interests/personal relationships which may be considered as potential competing interests: J‐F.G. reports a relationship with Amplitude SAS that includes consulting or advisory. The other authors (M.P., S.S., I.R.G., V.S., R.F., G.M.) declare that they have no known competing financial interests or personal relationships that could have appeared to influence the work reported in this article.
